# Who’s at Risk? Gauging Susceptibility to Air Pollutants

**DOI:** 10.1289/ehp.119-a176a

**Published:** 2011-04

**Authors:** M. Nathaniel Mead

**Affiliations:** **M. Nathaniel Mead**, a science writer living in Durham, NC, has written for *EHP* since 2002

What makes one individual more susceptible than another to the harmful effects of air pollution? If particulate matter (PM) differentially affects certain populations, which factors or characteristics are most likely to account for the heightened risk? These are the questions behind a new review of studies that examined various characteristics linked with susceptibility **[*EHP*** **119(4):446–454; Sacks et al.]**.

To assess the differential effects of PM on specific populations, epidemiologic studies often conduct stratified analyses; a stronger association between PM and the health effect being measured in one subgroup compared with another provides evidence for a more susceptible population. Additional insight can be gleaned via controlled human exposure studies (which examine individuals with a preexisting disease) and toxicologic studies using animal models of disease. The authors of the current study integrated these various lines of evidence to determine whether there was coherence of associations across the scientific disciplines. They also assessed the biological plausibility of specific characteristics identified in epidemiologic studies as potentially conferring susceptibility to PM-related health effects.

The authors focused on the collective evidence evaluated in the most recent science review of the PM National Ambient Air Quality Standards and also built upon the evidence presented in previous reviews. The studies examined the health effects primarily due to both short- and long-term exposures to the fine and/or coarse fractions of PM.

Overall, the characteristics of populations most associated with increased susceptibility to PM-related health effects included 1) life stage, specifically children and older adults; 2) preexisting cardiovascular and respiratory diseases; 3) specific genetic polymorphisms; and 4) low socioeconomic status, as measured by educational attainment and income. The authors found more limited evidence suggesting an increase in PM-related health effects in individuals with diabetes, chronic obstructive pulmonary disease, and increased body mass index. Potentially increased risks of PM-related health effects by sex and race/ethnicity also were indicated, although these associations were not consistent across health effects, PM size fractions, or, in some instances, study locations.

The authors concede they are unable to clearly state the overall strength of the evidence for some characteristics of potentially susceptible populations due to inconsistent evidence across epidemiologic studies as well as lack of information from experimental studies regarding biologically plausible mechanisms.

However, the novel integrative approach used to identify characteristics of populations potentially susceptible to PM may be a valuable assessment tool for other air pollutants. The authors also propose a comprehensive definition of “susceptibility” to encompass all populations potentially at increased risk of adverse health effects as a consequence of exposure. Use of such a standardized definition could help reverse inconsistencies in terminology within the epidemiologic literature that may have complicated the identification of high-risk populations to date.

## Figures and Tables

**Figure:**
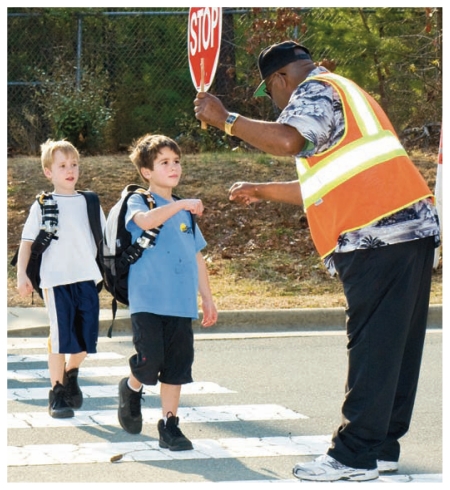
Biomonitoring studies have shown that children are especially vulnerable to the health effects of PM exposure.

